# Age-related trends of inhibitory control in Stroop-like big–small task in 3 to 12-year-old children and young adults

**DOI:** 10.3389/fpsyg.2014.00227

**Published:** 2014-03-18

**Authors:** Yoshifumi Ikeda, Hideyuki Okuzumi, Mitsuru Kokubun

**Affiliations:** ^1^Department of Special Needs Education, Faculty of Education, Tokyo Gakugei UniversityTokyo, Japan; ^2^Japan Society for the Promotion of ScienceTokyo, Japan

**Keywords:** inhibition, executive function, cognitive control, day–night task, Stroop

## Abstract

Inhibitory control is the ability to suppress competing, dominant, automatic, or prepotent cognitive processing at perceptual, intermediate, and output stages. Inhibitory control is a key cognitive function of typical and atypical child development. This study examined age-related trends of Stroop-like interference in 3 to 12-year-old children and young adults by administration of a computerized Stroop-like big–small task with reduced working memory demand. This task used a set of pictures displaying a big and small circle in black and included the same condition and the opposite condition. In the same condition, each participant was instructed to say “big” when viewing the big circle and to say “small” when viewing the small circle. In the opposite condition, each participant was instructed to say “small” when viewing the big circle and to say “big” when viewing the small circle. The opposite condition required participants to inhibit the prepotent response of saying the same, a familiar response to a perceptual stimulus. The results of this study showed that Stroop-like interference decreased markedly in children in terms of error rates and correct response time. There was no deterioration of performance occurring between the early trials and the late trials in the sessions of the day–night task. Moreover, pretest failure rate was relatively low in this study. The Stroop-like big–small task is a useful tool to assess the development of inhibitory control in young children in that the task is easy to understand and has small working memory demand.

## INTRODUCTION

Inhibitory control is the ability to suppress competing, dominant, automatic, or prepotent cognitive processing at perceptual, intermediate, and output stages (Nigg, 2000; Friedman and Miyake, 2004; see Ikeda et al., 2013a, for a discussion of classification in inhibitory control). That ability is used when the cognitive processing must be suppressed merely because it is inappropriate and when the cognitive processing must be suppressed in favor of a subdominant but appropriate one. Inhibitory control has been suggested as playing a critical role in executive function: higher order cognitive function that coordinates a goal-directed behavior (Harnishfeger and Bjorklund, 1994; Miyake et al., 2000; Anderson, 2001, 2002; Miyake and Friedman, 2012). Deficits in inhibitory control have also been implicated in behavioral problems associated with developmental disorders such as attention deficit hyperactivity disorder (ADHD) (Barkley, 1997; Ozonoff and Jensen, 1999; Friedman and Miyake, 2004; Spronk et al., 2008; Song and Hakoda, 2011; Yasumura et al., 2014). Inhibitory control is a key cognitive function of typical and atypical child development.

Inhibitory control is often measured in young children by administering the Stroop-like day–night task (Gerstadt et al., 1994; for a review, see Montgomery and Koeltzow, 2010). In the day–night task, children are presented with either a day picture of the sun or a night picture of the moon and stars, and they are instructed to say “day” to the night picture and “night” to the day picture. During the task, children must (a) suppress a dominant response of naming what a picture represents and (b) execute a competing subdominant response based on the instructions. Previous reports have described that performance of the day–night task improves significantly in young children (Montgomery and Koeltzow, 2010). Gerstadt et al. (1994) reported that the Stroop-like interference, measured as the difference of response time (RT) between experimental (saying the opposite of what is shown for day/night cards) and control conditions (saying “day” or “night” to abstract shapes), decreases in children between ages 3.5 and 5. Accuracy also improves concomitantly with age in children aged 3–7. Recent studies confirmed these findings, using variants of the day–night task with a range of stimuli and responses, such as color labels and basic-level object names (e.g., Simpson and Riggs, 2005a,b).

Difficulty in the day–night paradigm is believed to arise because of response competition occurring within the response set during testing. Although it was expected that the stronger association between word pairs makes the task more inhibitory demanding, recent research has demonstrated that what causes prepotency of response is not the relation between the response-to-be activated and the response-to-be suppressed (Diamond et al., 2002) but membership in the response set (Simpson and Riggs, 2005a). The problem is that the correct response on one trial is also the incorrect but prepotent response on subsequent trials. The potency of the incorrect response is magnified on each trial because of its activation during testing (by virtue of its inclusion in the response set) coupled with its depiction throughout the testing. In other words, the structure of the day–night task elevates response competition because (a) the response alternative that must be suppressed is depicted on the test card (e.g., “night” for the night card) and (b) the incorrect response alternative was previously activated on previous trials (e.g., “night” for day cards) in the case of a correct response, i.e., a response set effect (Simpson and Riggs, 2005a; Montgomery et al., 2008; Simpson et al., 2012).

Working memory is also presumed to be an important factor related to the performance in the day–night paradigm. Working memory may be involved because resolving which of the conflicting responses must be suppressed entails holding the task rules in mind (“say ‘day’ for night card” and “say ‘night’ for day card”). In fact, some studies report deterioration in performance occurring between the first four trials and the last four trials in the sessions of the day–night task in young children (e.g., Gerstadt et al., 1994). These reports suggest that young children may have forgotten rules or that working memory demands add to the processing requirements of the task and consequently compromise inhibitory mechanisms (Montgomery and Koeltzow, 2010). Then, it is expected to reduce the working memory demands in the day–night paradigm so that the task primarily measures inhibitory control.

Working memory demands may also be related to learning the task rules. It might be true that the day–night task recruits working memory to a certain extent because learning the combination between words and pictures (“day” for a night picture of the moon and stars and “night” for a day picture of the sun) is not easy to understand for young children. Actually, previous studies with the day–night task had a great pretest failure rate, especially in children aged between 3 and 4 (e.g., Gerstadt et al., 1994). A problem is that with more children failing the pretest, the sample of children whose RT and accuracy data are analyzed is not representative of the population. Probably, the children with the poorest inhibitory control get excluded.

This study was conducted to examine age-related trends of inhibitory control in various age groups by administration of a Stroop-like day–night variant with reduced working memory demand. For this study, 3 to 12-year-old children and young adults were administered a Stroop-like big–small task. The task used a set of pictures displaying a big or small circle in black and required participants to produce sized-based responses following instructions given by the experimenter. The size labels “big and small” were used because they are well understood even by very young children and because they are distinctive and opposite, both of which may facilitate learning and holding the task rules. The task has two conditions: the opposite condition, in which a participant says the opposite of what is shown with card pairs, and the same task condition, in which a participant simply names what the stimulus represents. Because the original study (Gerstadt et al., 1994) used a different combination of words and pictures in the opposite condition (saying the opposite of what is shown for day–night cards) to that used in the control condition (saying “day” or “night” to abstract shapes), the degree to which a picture evokes a particular response was not controlled. Comparison between opposite and same conditions, as in this study, indicates the inhibitory processes, controlling for a difference in the degree to which a picture evokes a particular response. In this study, unlike the standard “card” version of the day–night task, the task was computerized to evaluate the correct RT more precisely.

## MATERIALS AND METHODS

### PARTICIPANTS

Participants were 113 typically developing people who were divided into six age groups: (a) 3–4 year, 20 children (10 boys, 10 girls; M age = 51.5 months, age range = 43–59); (b) 5–6 year, 14 children (7 boys, 7 girls; M age = 68.6 months, age range = 60–83); (c) 7–8 year, 20 children (11 boys, 9 girls; M age = 95.1 months, age range = 87–107); (d) 9–10 year, 19 children (9 boys, 10 girls; M age = 119.6 months, age range = 108–131); (e) 11–12 year, 17 children (9 boys, 8 girls; M age = 144.4 months, age range = 133–153); and (f) 23 young adults (9 men, 14 women; M age = 21.1 year, age range = 18–24). Children were recruited through local mainstream preschool and elementary school programs. Young adults were recruited from a university. All participants speak Japanese as a first language. Criteria for inclusion were the absence of bilingualism and absence of behavioral or educational problems, which would affect the study of inhibitory control.

### MATERIALS AND METHODS

The Stroop-like big–small task used a set of pictures displaying either a big (12 cm diameter) or a small (1 cm diameter) circle in black. The same set of pictures was used in the same and opposite conditions. SuperLab (Cedrus Corp., San Pedro, CA, USA) controlled the task, presenting stimuli and recording participants’ vocal responses (error and time).

### PROCEDURES

Participants were tested individually in quiet rooms at their respective schools. At arrival, a participant was asked to be seated next to the experimenter at the table and approximately 50 cm in front of a monitor with a headset microphone. Subsequently, the experimenter explained that they were going to play a “game” in which they would see two pictures. The experimenter showed the participant the big and small circles at the same time on the screen and asked him or her to point to the big circle and the small circle in turn. All participants were able to do this. Then, each was administered the Stroop-like big–small task. In this task, the same condition was arranged to precede the opposite condition in an attempt to elicit robust interference.

Prior to the test phase, participants were trained on how to play each “game.” For the same condition, the experimenter said, “Here is a picture of big circle (show a big circle on the monitor). When this picture is shown, I want you to say ‘big’. And, here is a picture of small circle (show a small circle on the monitor). When this picture is shown, I want you to say ‘small’.” The participant did four practice trials (big–small–small–big). If the participant made any error, then the participant was corrected, reminded of the rules, and administered another four practice trials. The task did not commence until the participant was 100% correct for a set. For the opposite condition, the experimenter said, “We are going to play an opposite game. Here is a picture of big circle (show a big circle on the monitor). When this picture is shown, I want you to say ‘small’. And, here is a picture of small circle (show a small circle on the monitor). When this picture is shown, I want you to say ‘big’.” The opposite condition required participants to inhibit the prepotent response of saying the same, a familiar response to a perceptual stimulus. The practice trials were identical to the same condition.

During the test phase, the participant was asked to respond as quickly and accurately as possible to a series of 20 stimuli (10 big circles and 10 small circles) for each task condition. All stimuli were presented one at a time and randomly at the center of the white screen on the monitor. At the instant a participant’s voice key was input, each stimulus was replaced by a fixation cross until the participant was judged by the experimenter to be ready to proceed to the next trial, looking at the fixation cross. The interstimulus interval was not controlled by SuperLab, as it was in a card version of the task, because some younger children have difficulty engaging in the task continuously. No feedback reminding participants of the task rules was given during testing.

### ANALYSIS

Numbers of errors and RT for correct responses were recorded. Trials were counted as incorrect when participants’ initial responses were errors, even if they self-corrected. The RT was measured as the interval in milliseconds between the presentation of a stimulus and the onset of the participant’s vocal response by the microphone. Analysis of RT was conducted only for the correct response. Mean and standard deviations of error rates and correct RT on the whole trials were calculated for each task condition. To examine changes of performance over the course of a session for each task condition, mean and standard deviations of error rates and correct RT were calculated for the first five trials and the last five trials, respectively.

The data were analyzed using analysis of variance (ANOVA). Specifically, two-way ANOVAs with the within-participant factor of condition (same and opposite) and between-participant factor of age group (3 to 4-year olds, 5 to 6-year olds, 7 to 8-year olds, 9 to 10-year olds, 11 to 12-year olds, and young adults) were conducted for error rates and correct RT. Also, three-way ANOVAs with the within-participant factors of condition (same and opposite) and serial position (first five trials and last five trials) and between-participant factor of age group (3 to 4-year olds, 5 to 6-year olds, 7 to 8-year olds, 9 to 10-year olds, 11 to 12-year olds, and young adults) were conducted for error rates and correct RT. Software was used for statistical analyses (SPSS 19.0 for Windows; SPSS Japan Inc., Tokyo, Japan). Unless otherwise noted, a 0.05 level of significance was adopted for all statistical analyses.

### ETHICAL APPROVAL

Informed consent was obtained from all adult participants and from a parent of each child participant before the assessment session. Our experimental protocol was administered in accordance with the guidelines of the Declaration of Helsinki and was approved by the institutional review board.

## RESULTS

### TREATMENT OF UNUSED DATA

An additional 14 participants were tested. Data from 9 participants were not included in this study because they showed results more than 3 SD from the mean of each age group (i.e., outliers). One 3-year-old child was not able to pass a pretest for the saying-opposite condition. Another 3-year-old child was not able to complete the task because his voice was too small to record. Three school-age children were excluded because of experimental error in recording the data.

### ACCURACY OF RESPONSE

**FIGURE 1:**
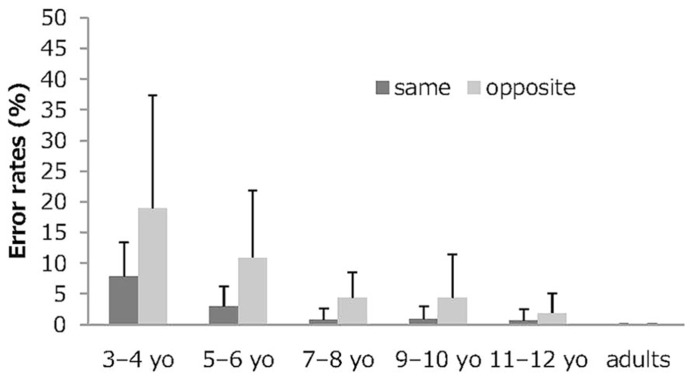
**Mean error rates in the Stroop-like big–small.** Error bars represent standard deviations. No adults showed an error.

**Figure 1** depicts the mean and standard deviations for the error rates in the Stroop-like big–small task by age group and condition. A 6 (age group) × 2 (condition) mixed-model ANOVA was conducted of the error rates. The analysis showed significant main effects for the age group (*F*_5,107_ = 18.40, *p*<0.001, partial η^2^ = 0.46), for condition (*F*_1,107_ = 26.73, *p* <0.001, partial η^2^ = 0.20), and for interaction of age group and condition (*F*_5,107_ = 4.02, *p* <0.01, partial η^2^ = 0.16). *Post hoc* Bonferroni tests yielded significant differences between-age group comparisons between 3 to 4-year olds and other age groups and between 5 to 6-year olds and other age groups for each condition (*p* <0.05). For each condition, between-age group comparisons among 7 to 8-year olds, 9 to 10-year olds, and 11 to 12-year olds and young adults were not significant. *Post hoc* Bonferroni tests also yielded significant differences between conditions for 3 to 4-year olds and 5 to 6-year olds (*p* <0.05), but not for other age groups (7 to 8-year olds, *p* = 0.084; 9 to 10-year olds, *p* = 0.107; 11 to 12-year olds, *p* = 0.588; young adults, *p* = 1.00). These results clarified that the interaction between age group and condition reflected age-related convergence of error rates in the same condition and in the opposite condition.

**FIGURE 2:**
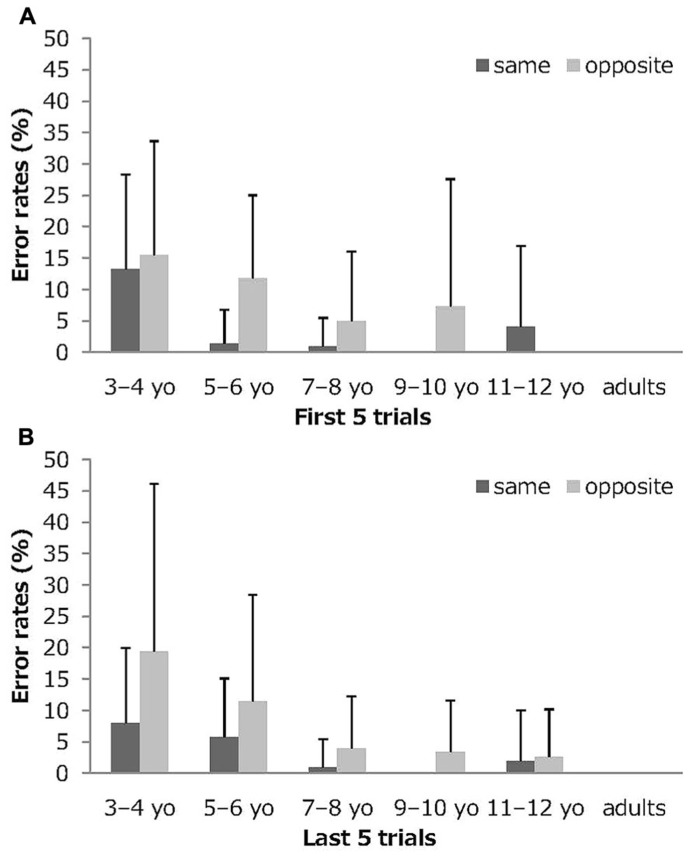
**Mean error rates early and late in the session in the Stroop-like big–small.** **(A)** mean error rates on the first five trials, **(B)** mean error rates on the last five trials. Error bars represent standard deviations. On the first five trials, no error was shown for 9 to 10-year olds in the same condition, for 11 to 12-year olds in the opposite condition, and for adults in the same and opposite conditions. On the last five trials, no error was shown for 9 to 10-year olds in the same condition and for adults in the same and opposite conditions.

**Figure 2** depicts the mean and standard deviations for the error rates early (the first five trials) and late (the last five trials) in the session in the Stroop-like big–small task by age group and condition. A 6 (age group) × 2 (condition) × 2 (serial position) mixed-model ANOVA was conducted of the error rates. The analysis showed significant main effects for the age group (*F*_5,107_ = 18.33, *p *<0.001, partial η^2^ = 0.46), for condition (*F*_1,107_ = 15.97, *p* <0.001, partial η^2^ = 0.13), and for interaction of age group and condition (*F*_5,107_ = 2.84, *p* <0.05, partial η^2^ = 0.12). Main effect for the serial position, the other two-way interactions, and the three-way interaction were not significant. These results clarified that there were no deterioration of performance over the course of a session in terms of the error rates. 

### CORRECT RESPONSE TIME

**FIGURE 3:**
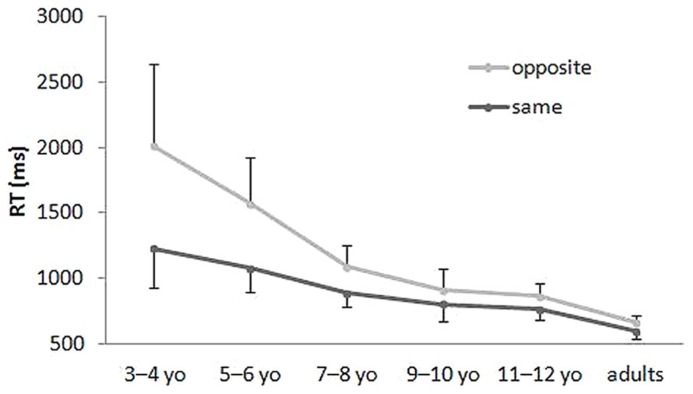
**Mean RT in the Stroop-like big–small.** Error bars represent standard deviations.

**Figure 3** depicts the mean and standard deviations for the correct RTs in the Stroop-like big–small task by age group and condition. A 6 (age group) × 2 (condition) mixed-model ANOVA was conducted of the correct RT. The analysis showed significant main effects for age group (*F*_5,107_ = 53.77, *p* <0.001, partial η^2^ = 0.72), for condition (*F*_1,107_ = 203.02, *p* <0.001, partial η^2^= 0.66), and for interaction of the age group and condition (*F*_5,107_ = 34.37, *p* <0.001; partial η^2^ = 0.62). *Post hoc* Bonferroni tests yielded all significant between-age group comparisons (*p* <0.05), except for between 7 to 8-year olds and 9 to 10-year olds and between 9 to 10-year olds and 11 to 12-year olds, for each condition. *Post hoc* Bonferroni tests also yielded significant differences between conditions for 3 to 4-year olds, 5 to 6-year olds, 7 to 8-year olds, and 9 to 10-year olds (*p* <0.05), but not for other age groups. Results clarified that the interaction between age group and condition reflected age-related convergence of error rates in the same condition and in the opposite condition.

**FIGURE 4:**
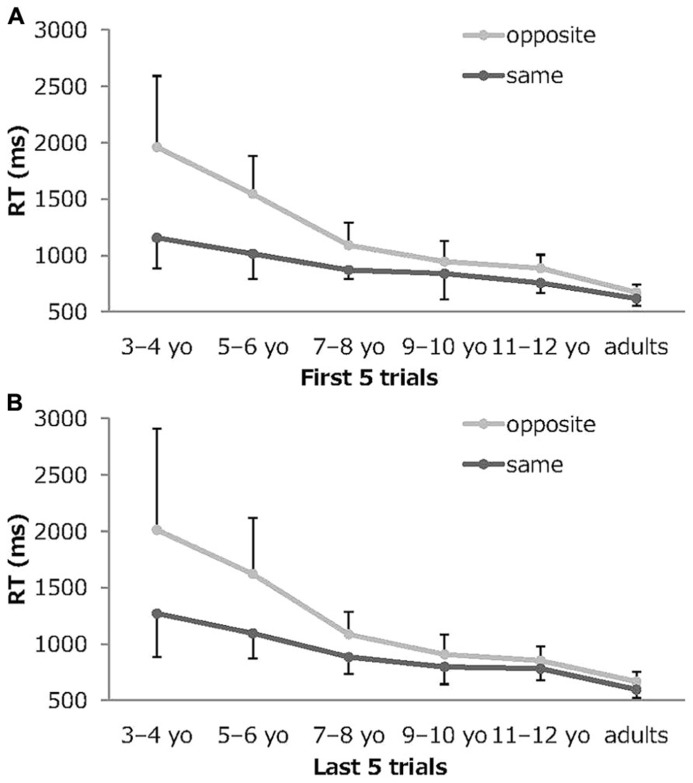
**Mean RT early and late in the session in the Stroop-like big–small.** **(A)** mean RT on the first five trials, **(B)** mean RT on the last five trials. Error bars represent standard deviations.

**Figure 4** depicts the mean and standard deviations for the correct RTs early (the first five trials) and late (the last five trials) in the session in the Stroop-like big–small task by age group and condition. A 6 (age group) × 2 (condition) × 2 (serial position) mixed-model ANOVA was conducted of the correct RT. The analysis showed significant main effects for the age group (*F*_5,107_ = 46.25, *p *<0.001, partial η^2^ = 0.68), for condition (*F*_1,107_ = 153.51, *p* <0.001, partial η^2^ = 0.59), and for interaction of age group and condition (*F*_5,107_ = 25.24, *p* <0.001, partial η^2^ = 0.54). Main effect for the serial position, the other two-way interactions, and the three-way interaction were not significant. These results clarified that there were no deterioration of performance over the course of a session in terms of the correct RTs.

## DISCUSSION

This study examined age-related trends of Stroop-like interference in 3 to 12-year-old children and young adults by administration of a computerized Stroop-like big–small task. In this study, the differences between the opposite and same conditions were compared among age groups for error rates and correct RT. It was hypothesized that working memory demand is reduced in the Stroop-like big–small task.

Results show that Stroop-like interference decreased markedly in children. The difference between conditions in error rates was significant for 3 to 4-year olds and 5 to 6-year olds but not for the older age groups although there were trends toward significance for some older age groups, which may be due to relatively small sample size. These results are consistent with the results obtained from previous studies using the day–night task and other variants of this task (Gerstadt et al., 1994; Simpson and Riggs, 2005b). However, this difference in correct RT was significant for 3 to 4-year olds, 5 to 6-year olds, 7 to 8-year olds, and 9 to 10-year olds. This result is consistent with the results reported by Simpson and Riggs (2005b), which used five age groups (3.5-, 5-, 7-, 9-, and 11-year olds) and reported that Stroop-like interference was greatest in 3.5-year olds, greatly reduced in 5-year olds, and thereafter declined more moderately up to the age of 11. In this study, the difference between 9 to 10-year olds and young adults were not significant, although a decrement of interference between older children and young adulthood was often observed in the Stroop color-word task (Ikeda et al., 2011, 2013b) and the Stroop-like task (Ikeda et al., 2013a). This decrement can be interpreted as reflecting reduced inhibitory demand in the Stroop-like big–small task compared to other inhibitory tasks.

This study used a variant of the day–night task particularly addressing the concept of size, “big” and “small.” These sizes were concrete for participants in this study because they were perceptual features of the stimuli that were used, which seemed to facilitate sampling of young children, having them feel more comfortable by learning and holding the rules in mind. Actually, fewer children refused participation or were unable to pass the pretest, compared to those of the original study using the day–night task (Gerstadt et al., 1994). A problem for previous research is that with more children failing the pretest, the sample of children whose RT and accuracy data are analyzed is not representative of the population. Moreover, the results showed no difference in error rates and correct RTs between the first five trials and the last five trials in the session, suggesting that participants did not forget the rules or that working memory recruited in the task did not compromise inhibitory mechanisms.

In conclusion, this study demonstrated that the difference between naming what stimuli represent and naming of the “opposite” of the stimuli was decreased significantly during young childhood in the Stroop-like big–small task that has smaller working memory demands than the original version of the day–night task. In other words, this study showed that inhibitory control develops rapidly in young children. The Stroop-like big–small task is a useful tool to investigate the development of inhibitory control in young children in that the task is easy to understand and has small working memory demand.

## Conflict of Interest Statement

The authors declare that the research was conducted in the absence of any commercial or financial relationships that could be construed as a potential conflict of interest.
